# Machine-learning techniques for quantifying the protolith composition and mass transfer history of metabasalt

**DOI:** 10.1038/s41598-022-05109-x

**Published:** 2022-01-26

**Authors:** Satoshi Matsuno, Masaoki Uno, Atsushi Okamoto, Noriyoshi Tsuchiya

**Affiliations:** Graduate School of Environmental Studies, Tohoku University, 6-6-20 Aza-Aoba, Aramaki, Aobaku, Sendai, 980-8579 Japan

**Keywords:** Geochemistry, Petrology

## Abstract

The mass transfer history of rocks provides direct evidence for fluid–rock interaction within the lithosphere and is recorded by compositional changes, especially in trace elements. The general method adopted for mass transfer analysis is to compare the composition of the protolith/precursor with that of metamorphosed/altered rocks; however, in many cases the protolith cannot be sampled. With the aim of reconstructing the mass transfer history of metabasalt, this study developed protolith reconstruction models (PRMs) for metabasalt using machine-learning algorithms. We designed models to estimate basalt trace-element concentrations from the concentrations of a few (1–9) trace elements, trained with a compositional dataset for fresh basalts, including mid-ocean ridge, ocean-island, and volcanic arc basalts. The developed PRMs were able to estimate basalt trace-element compositions (e.g., Rb, Ba, U, K, Pb, Sr, and rare-earth elements) from only four input elements with a reproducibility of ~ 0.1 log_10_ units (i.e., ± 25%). As a representative example, we present PRMs where the input elements are Th, Nb, Zr, and Ti, which are typically immobile during metamorphism. Case studies demonstrate the applicability of PRMs to seafloor altered basalt and metabasalt. This method enables us to analyze quantitative mass transfer in regional metamorphic rocks or alteration zones where the protolith is heterogeneous or unknown.

## Introduction

The mass transfer history of rocks provides direct evidence for fluid–rock interactions within the lithosphere, including seafloor alteration, subduction zone metamorphism, geothermal fluid activity, and fault zone processes. In particular, trace elements are sensitive to fluid–rock interactions and record such interactions by changing compositions in the rocks or fluids. Mass transfer analyses in the context of subduction-related metamorphism reveal trace-element transport via dehydration reactions in the subducting slab^1–3^ and element cycling in the subduction zone^4,5^ that is chemically linked to arc basalt^6,7^. Seawater reacts with oceanic crust and transfers trace elements through weathering and hydrothermal vents^8–11^. The transfer of trace elements also reflects dynamic fluid–rock interactions, such as pulsed fluid flow related to seismic events^2,12–16^. Therefore, analyses of mass transfer in chemically altered rocks are essential for understanding fluid-related processes within the lithosphere and the evolution of surface environments.

The general method of mass transfer analysis is to compare the composition of the protolith/precursor with that of metamorphosed or altered rock. Mass transfer at the outcrop scale (< 100 m) can be estimated by comparing the compositions of altered rock with that of adjacent unaltered host rock^17–22^. At larger scales > 1 km (e.g., comparisons of rocks in different metamorphic belts), mass transfer can be qualitatively evaluated by comparing chemical differences between metamorphosed rocks (e.g., metabasalt) and their likely protoliths^4,5^ (e.g., mid-ocean ridge basalt or MORB). Mass transfer can also be estimated by comparing mobile/immobile elemental ratios (e.g., K/Th) between samples and their likely protoliths. In such analyses, the choice of the likely protolith composition depends on the knowledge of trained geochemists and/or further subjective observations, and the estimated amount of mass transfer may therefore vary among researchers.

In many cases, the major challenge in mass transfer analysis is that we cannot access the exact protolith of metamorphosed/altered rocks, except for cases where the protoliths are evident in outcrop. As the spatial variations in protolith (e.g., basalt and sediment) composition are generally large^23–26^, it is difficult to quantitatively evaluate the amount of mass transfer for each sample. Recent analyses of regional metamorphic belts have also revealed that protoliths of metamorphic rocks differ in their depositional ages and tectonic setting among different units or metamorphic grades of rock^5,27–30^, suggesting that it is unrealistic to assume a uniform protolith composition in regional metamorphic belts or alteration zones. Therefore, to quantify mass transfer more precisely, it is necessary to estimate the protolith composition for individual samples.

Natural observations and experiments have revealed that the intensity of mass transfer varies with the elements involved, pressure, temperature, and fluid chemistry. Large-ion lithophile elements (LILEs; e.g., Rb, Ba, and Sr) are subject to large mass transfer during seafloor alteration and during metamorphism because they are highly soluble in metamorphic fluids^10,31–33^. Analyses of mineral veins and alteration zones have confirmed the mobility of these elements during metamorphism^1,3,34^. Other elements, such as high-field-strength elements (HFSEs), generally show little mass transfer during seafloor alteration^10,35,36^ and have low solubility under the typical pressure–temperature conditions of metamorphism^31–33^. Consequently, they are generally considered to be “immobile”^4,5,10,35–37^. Compilations of mass transfer under various metamorphic conditions and in a range of environments have suggested that the mobility of HFSEs decreases roughly in the order of rare-earth elements (REEs) > U > Nb > Ti > Th = Zr for high-pressure subduction zone environments^37^. These elements are widely considered immobile elements and are therefore used to discriminate the tectonic setting of metabasalt^38,39^. The general success of discrimination diagrams indicates that immobile elements retain information on the protolith. Provided that there are generally multidimensional correlations among trace-element compositions in basalt^26,40^, it should be possible to formulate the relationships between immobile elements and potentially mobile elements in basalt. This would enable us to reconstruct protolith compositions from concentrations of immobile elements in metamorphosed or altered basaltic rocks.

Advances in data science have provided excellent tools for extracting information from large amounts of multidimensional data. In particular, machine learning can identify complex patterns in images and extract information from multidimensional table data. The recent increase in the amount of data in geochemical compositional databases (e.g., PetDB and Georock) has made machine-learning modeling possible for geochemical research^26,41^. For example, machine learning has been successfully applied to discriminate the tectonic setting of basalt from geochemical data^26^ and classify metamorphic protoliths from major element data^42^. Machine-learning algorithms have also been used to estimate the chemical composition of the protolith of hydrothermally altered volcanic rock^43^, showing that machine learning is also effective for regression problems involving the chemical compositions of rocks.

In this study, we focus on modeling the trace-element concentrations in the protolith of metabasalt, with the aim of reconstructing the mass transfer in regional metamorphic rocks or alteration zones where the protolith is heterogeneous or unknown. Metabasalt was chosen as the target because basalt is one of the major components of oceanic crust, and subducting slab and is an important source of trace elements in metamorphic and hydrothermal fluids^1,3,9,44,45^. In addition, compositional variations in basalt are relatively simple compared with those in sediments and other volcanic rocks, and are suitable to model as a first trial of the approach. We focus on the reconstruction of trace elements, and do not focus on systems with substantial addition or removal of major elements.

We develop protolith reconstruction models (PRMs) to estimate the protolith composition of metabasalt using machine learning. First, using a basalt whole-rock compositional dataset, we develop empirical models that learn multi-elemental correlations among the dataset. The models estimate the trace-element compositions of basalt based on the concentrations of a few (one to nine) elements. We determined the numbers and combinations of input elements needed to precisely predict the output concentrations. Results show that basalt trace-element concentrations (i.e., Rb, Ba, U, K, Pb, Sr, and REEs) can be estimated from data of only four elements (i.e., Th, Nb, Zr, and Ti). Finally, we apply the selected four-element PRMs to altered seafloor basalt and metabasalt, and demonstrate the validity of the model and provide examples of mass transfer analyses for metamorphic rocks.

## Model description

### Overview of PRMs

**Figure 1:**
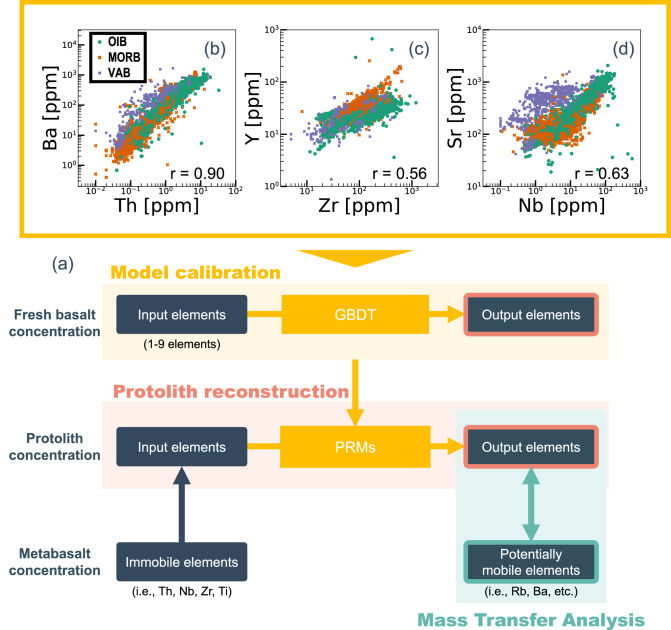
(**a**) Schematic overview of protolith reconstruction models (PRMs). Empirical models were calibrated using the protolith (basalt) compositional dataset and applied to metabasalt concentrations. Assuming that the concentrations of immobile elements in metabasalt are identical to those in protolith basalt, these concentrations can be assigned as inputs and used to reconstruct protolith concentrations. Finally, the mass transfer history is evaluated by comparing the concentrations of potentially mobile elements between the metabasalt and reconstructed protolith. (**b**–**d**) Distribution of the compositional dataset used in this study (compiled from the geochemical database at https://search.earthchem.org/). (**b**) Th and Ba, (**c**) Zr and Y, and (**d**) Nb and Sr.

The PRMs were developed through a machine learning analysis of a dataset of basalt geochemical data. The models are used to estimate concentrations of particular trace elements (i.e., potentially mobile elements) from combinations of HFSEs (i.e., potentially immobile elements) (Fig. 1a), based on an empirical approach. The PRMs were calibrated using a dataset for fresh basalt.

In the metabasalt, it could be assumed that the concentrations of immobile elements are identical to those of its protolith, provided that the rock has not been subject to substantial addition or removal of mass. The limitation of this assumption is discussed in the subsections below entitled “Assumptions of PRMs in their application to metabasalt” and “Limitations of PRMs in their application to metabasalt”. Adopting this assumption, we can reconstruct the composition of the protolith basalt by applying the PRMs to immobile element data. The mass transfer history is evaluated by comparing the concentrations of potentially mobile elements between the metabasalt and reconstructed protolith (Fig. 1a).

### Basalt dataset

Basalt compositional data were taken from the PetDB database (https://search.earthchem.org/). The data were selected in terms of potential protoliths of metabasalt in metamorphic terrains and ophiolites, and include mid-ocean ridge basalt (MORB), ocean-island basalt (OIB), and volcanic arc basalt (VAB). Data of 8422 fresh basalt samples were compiled to assess the trace-element concentrations of 16 elements (Rb, Ba, U, K, La, Ce, Pb, Sr, Nd, Y, Yb, Lu, Zr, Th, Ti, and Nb). The dataset includes some erroneous data (e.g., typographical errors), as well as data for weathered basalt samples whose compositions may have been significantly modified from those of fresh samples. Accordingly, we corrected and filtered the data on the basis of the following criteria, partly following previous studies (e.g., Trépanier et al.^43^).

1. Data distributions were checked on 2D scatter plots for erroneous records. In cases where there were significant outlier data compared with the rest of the dataset, we checked the original paper and corrected the data if possible.
2. Samples with LOI values of > 2.5 wt% were considered as altered (according to the IUGS volcanic classification; Le Maitre et al.^46^) and were excluded from the analysis. A total of 2539 samples out of the 8422 samples of the dataset had LOI data, of which 327 samples were rejected.
3. Samples with a Chemical Index of Alteration (CIA^47^) value of > 50 were discarded. The CIA for fresh basalt is usually 35–50^48^. 31 samples were rejected from the 8422 samples of the dataset.

After filtering, we obtained 8080 basalt samples that were considered to represent fresh rock samples.

The distribution of compositional data for these basalts varies with the element of interest. Th and Ba contents have a relatively high correlation coefficient (r = 0.90), the correlation between Zr and Y varies with the type of basalt (r = 0.56), and there is a weak correlation between Nb and Sr (r = 0.63) (Fig. 1b–d). These data distributions suggest non-linear and multidimensional relationships among the concentrations of the 16 elements.

### Machine-learning algorithms

We selected the gradient boosting decision tree (GBDT) as the machine-learning algorithm. The GBDT is one of several decision tree algorithms capable of fitting complex datasets (i.e., non-linear structural data) and performing calculations with high speed and accuracy^49^. Compared with other machine-learning algorithms, such as Support Vector Machine (SVM) or Deep Learning, GBDT is the better choice for tabular data in terms of performance and parameter tuning^50^. We adopted the LightGBM algorithm because it has the lowest computational cost among GBDT algorithms^51^. We initially tested the various machine-learning algorithms such as SVM and multiple linear regressions (“Supplementary Information Note”). The results indicated that GBDT is the suitable algorithm in terms of computational cost, and accuracy.

### Calibration and evaluation of the models

The basalt compositional data were randomly divided into training and test data at a ratio of 4:1. The GBDT algorithm analyzed the training data and constructed models with K-fold cross-validation. During the K-fold cross-validation, data are further split into training subset and validation subset that are used to optimize hyperparameters^49^. Model performance was evaluated using the root mean squared error (RMSE) in log space between the estimated output and the measured data:

1 $${E}_{i} =\frac{1}{N} \sum_{j}^{sample}\sqrt{{\left({\mathrm{log}}_{10}{y}_{i,\mathit{ j}}^{\mathit{estimated}} -{\mathrm{log}}_{10}{y}_{i, j}^{test} \right)}^{2}}$$

where *E*_*i*_ is the RMSE for element *i*, *N* is the number of samples, *y*_*i,j*_^estimated^ is the estimated concentration of element *i* in sample *j*, and *y*_*i,j*_^test^ is the measured concentration of element *i* in sample *j*. We adopted Bayesian optimization for hyperparameter tuning of each model, and the optimal hyperparameters were searched by rebuilding the model 50 times, based on evaluation of validation data. See the “Methods” section for more details.

### Choice of input and output elements

The elements used as input and output were determined from the degree of mass transfer reported in previous studies. LILEs are mobile during fluid activity in subduction zones, contact metamorphism, and seafloor alteration, whereas HFSEs are relatively immobile during fluid activity^10,32,33,35–37^. The order of mobility of HFSEs is REEs > U > Nb > Ti > Th = Zr, as determined from observations of natural metamorphic rocks and experiments under a range of metamorphic conditions^37^.

As mentioned above, the number and combination of mobile and immobile elements cannot be uniquely determined and may vary substantially between different geochemical systems. The user must determine the appropriate number and combination of input elements when applying the PRMs to metabasalt, and mobile elements should not be used as input elements. To enable the application of the PRMs to various geochemical systems, we selected input and output elements as follows: the input elements were combinations of between 1 and 9 elements from Zr, Th, Ti, Nb, La, Ce, Nd, Yb, and Lu; and the output elements were Rb, Ba, U, K, La, Ce, Pb, Sr, Nd, Y, Yb, Lu, Zr, Th, Ti, and Nb. Elements used as input elements were not considered as output elements.

We first constructed models for all combinations of input and output elements. Each model is used to estimate an output element concentration from a combination of input element concentrations (e.g., input elements: Th, Nb, Ce; output element: Rb). Basalt compositional data were chosen to ensure that there were no missing values for input and output elements in the utilized dataset (typically 3000–5000 samples). The number of combinations of input elements is 2^9^ − 1 = 511. For each input element combination with *n* input elements, there are (16 − *n*) output elements. Accordingly, we developed $\sum_{n=1}^{9}\left\{{{}_{9}C}_{n} (16-n)\right\}=5872$ machine-learning models in total.

### Assumptions of PRMs in their application to metabasalt

The application of PRMs to metabasalt is limited to cases where it can be assumed that the concentrations of immobile elements are identical between the metabasalt and its protolith (Fig. 1a). For example, PRMs cannot be applied to systems with substantial addition or removal of major elements, as immobile element concentrations can change in response to the addition or removal of mass. To apply PRMs to metabasalt, the total mass gain or loss in the sample should be within the analytical uncertainty of trace-element concentrations (i.e., ± 10–20 wt% considering the reproducibility among different analytical methods or laboratories^52–55^).

## Results

**Figure 2:**
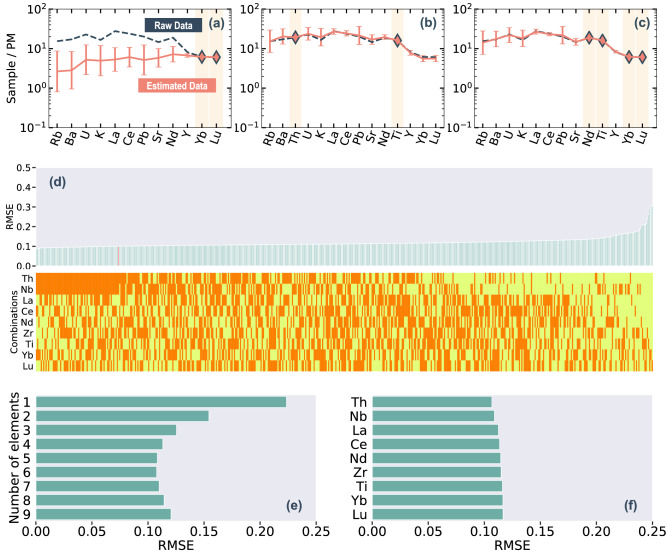
(**a–c**) Estimated primitive-mantle-normalized trace element concentrations in basalt. Pink diamonds indicate the input concentrations. Predicted data were obtained from the input concentrations of (**a**) Yb and Lu; (**b**) Th and Ti; and (**c**) Nd, Ti, Yb, and Lu. Raw basalt compositional data are shown as a dashed dark-blue line, and estimated basalt compositional data are shown as a pink line. Composition of the primitive mantle is from Sun and McDonough (1989). The error bar indicates root mean squared error (RMSE) for each model. (**d**) Average RMSE scores of all output elements for each combination of input elements (511 cases), and combinations of input elements for each model. In the upper plot, the red vertical line indicates the input combination of Th, Nb, Zr, and Ti. In the lower plot, the orange elements are used in combinations, and yellow elements are not used. (**e**) Average RMSE scores for all models using a particular number of input elements. (**f**) Average of all models containing a particular element as an input.

Figure 2a–c shows examples of the estimated compositions for a specific basalt sample, for different sets of input elements. The reproducibility of the estimation is dependent mainly on the choice of input elements. For example, in the case of the input elements being Yb and Lu, the reproducibility (i.e., the difference between the actual and estimated compositions) for each element is large (Fig. 2a; i.e., > 1 in log_10_ units). In contrast, for input elements of Th and Ti, or Nd, Ti, Yb, and Lu, the reproducibility for each element is greatly improved and is < 0.2 in log_10_ units (Fig. 2b,c).

The effect of the choice of input element was evaluated by taking the averages of RMSE scores. Figure 2d shows the average RMSE scores of all output elements for each combination of input elements (511 cases). The best model score was obtained using input elements of Th, Nb, Ce, and Yb (0.087) and the worst was obtained using an input element of Lu (0.30). The top 13.6% of models all include Th, and 14.6% of models include Nb. Figure 2e shows average RMSE scores for all models classed by the number of input elements. In the case of more than four input elements, the averaged RMSE scores converge around 0.11 (0.113 for four input elements, 0.108 for five input elements, and 0.107 for six input elements). In addition, we evaluated the effect of each input element by taking the average of all models containing a particular element as input (Fig. 2f). The average scores show little change with input elements compared with the number of elements. Models using Th and Nb as inputs have slightly lower average scores than the other models (0.106 for Th, 0.109 for Nb, and ~ 0.115 for the other elements).

The top 43% of models fall within the range of RMSE ≤ 0.11 (Fig. 2d). The three best models each have five input elements: Th, Ti, Nb, Ce, and Yb (RMSE = 0.089); Zr, Th, Nb, Ce, and Yb (RMSE = 0.091); and Th, Ti, Nb, La, and Yb (RMSE = 0.092). Among the models with four input elements, the best combinations are Th, Nb, Ce, and Yb (RMSE = 0.087); Th, Nb, Nd, and Yb (RMSE = 0.089); and Th, Nb, La, and Yb (RMSE = 0.091). The top 43% of models (221 combinations of input elements) have almost identical RMSE values (0.09–0.11), or reproducibilities of ± 0.09–0.11 in log_10_ units, or ± 23–28%.

## Discussion

### Dependence of model performance on input elements

The RMSE scores generally improve with an increasing number of input elements until there are more than four elements (Fig. 2e). This result indicates that the trace-element composition of basalt can be suitably estimated from only four (or five) input elements (i.e., RMSE ~ 0.11, or reproducibility of ± 28%). In addition, the RMSE score of all output elements does not change substantially with different combinations of input elements (i.e., the top 43% of models have RMSE = 0.09–0.11; Fig. 2d). Consequently, these results show that four (or five) input elements are sufficient for PRMs, and users can select those elements that best suit their specific cases (i.e., immobile elements in the geochemical system in question).

The model performance in estimating a particular output element improves when input elements have similar incompatibility to that of the output element. For example, the RMSE of Ce is improved with the input combination of La and Nd (Supplementary Fig. S1). The dependence of RMSE on input elements indicates that input elements with closer compatibility to that of the output element contain more identifying information on protolith composition. For example, the RMSE of Ce gradually improves when the input elements have closer compatibility with Ce^56^. Accordingly, to improve the overall estimation, it is necessary to choose input elements that have a wide range of incompatibility when combined.

### PRM reproducibility: the example of Th, Nb, Zr, and Ti as input elements

As a typical example of PRMs, we present PRMs with input elements of Th, Nb, Zr, and Ti. This element combination satisfies the four-element and wide-incompatibility element^56^ criteria for the input elements described above. In addition, they are the most immobile elements as judged from both natural observations and experiments^31–33,37^, and PRMs based on these elements should be among the most suitable models for application to metabasalt. This subsection discusses the results and reproducibility of test data. Case studies are presented to demonstrate the validity of PRMs and their application to mass transfer analyses.

**Figure 3:**
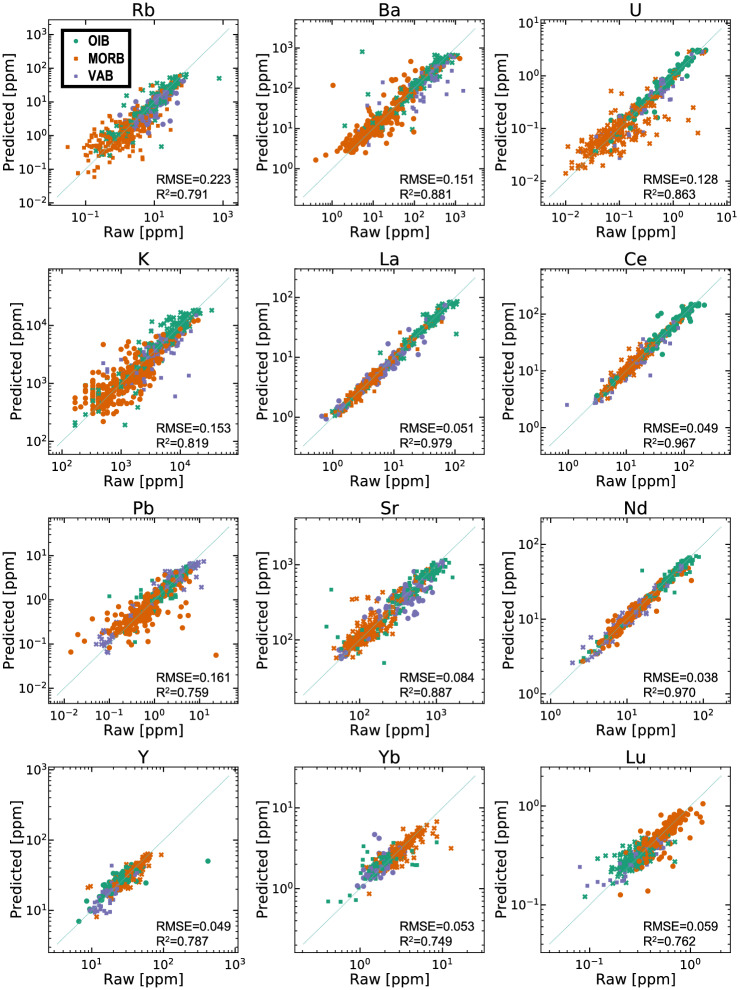
Scatter plots of raw (measured) concentrations versus predicted concentrations using the final PRMs with Th, Nb, Zr, and Ti as input elements. The PRMs were applied to test data of the basalt dataset, which covers three different tectonic settings (mid-ocean ridge basalt, ocean-island basalt, and volcanic arc basalt).

We applied the PRMs with input elements of Th, Nb, Zr, and Ti to the test data of the basalt compositional dataset. The PRMs were constructed using ~ 3000 basalt samples (i.e., data containing all of the input elements and an output element) and can estimate protolith compositions with an RMSE of ~ 0.1 (i.e., ± 25%; Fig. 2d). The estimated concentrations show largely linear relationships with the raw (measured) concentrations in log–log space (Fig. 3).

These results show that the PRMs closely reproduce individual elements through a wide range of their compositions. Scatter plots of La, Ce, Sr, Nd, Y, Yb, and Lu show relatively minor deviations (i.e., RMSE < 0.1) from the 1:1 line and almost no dependence on tectonic setting. In comparison, distributions of Rb, Ba, U, K, and Pb have relatively large dispersions (i.e., RMSE > 0.1). In particular, the K data are widely dispersed at low concentrations. These results also affect the distribution of reproducibility of each element (Fig. S2). The reproducibility of Rb, Ba, U, K, and Pb differs with tectonic setting, whereas the other elements show little or no dependence on tectonic setting: MORB has a wider range of reproducibility than OIB and VAB for Rb, U, K, and Pb. VAB has a wider range of reproducibility than OIB and VAB for Ba.

One explanation for the dependence of dispersion on element concentration is the analytical detection limit. In particular, the raw data for K have identical values for samples with low concentrations (≤ 10^3^ ppm), and such data show low reproducibility, probably because they are close to the detection limit of K in X-ray fluorescence (XRF) analyses or the resolution of the original dataset was coarse (i.e., ~ 0.1 wt.%). Although such low-concentration data could have been removed by filtering before modeling, the filtering of low-K samples would have limited the compositional diversity of the basalt data. Therefore, we incorporated these data into the training data.

An alternative explanation is seafloor alteration, for which Rb, Ba, U, K, and Pb are mobile^10,36,57^. Some samples of MORB and VAB might have already undergone mass transfer by hydrothermal alteration because parts of these were collected from the ocean seafloor, with the sample data being correspondingly affected. Although the basalt compositional dataset had been filtered for “fresh basalt”, there is a possibility that the filtering had not wholly rejected the altered basalt.

**Figure 4:**
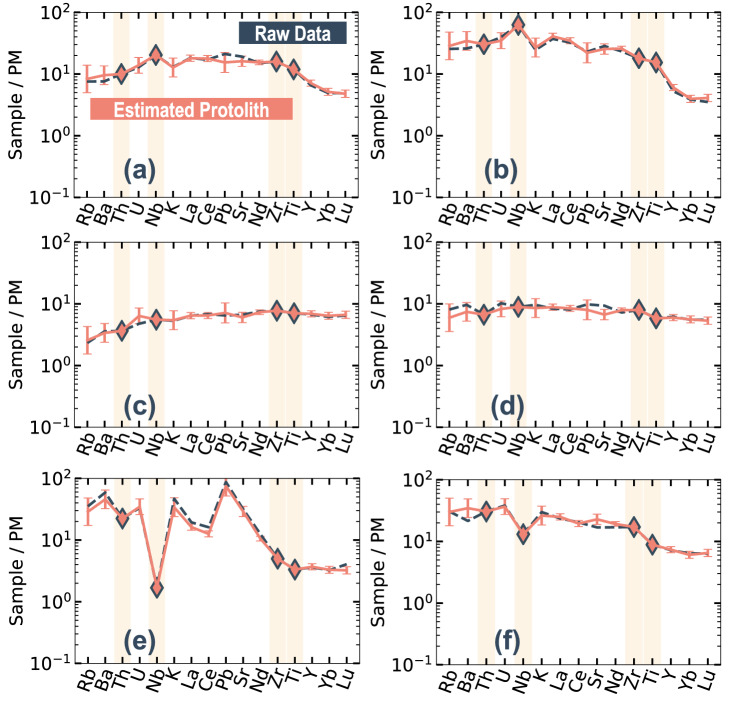
Primitive-mantle-normalized trace element concentrations of basalt estimated using the four-element PRMs with Th, Nb, Zr, and Ti as input elements. The error bar indicates root mean squared error (RMSE) for each model. Samples for each panel are examples of (**a**, **b**) OIB, (**c**, **d**) MORB, and (**e**, **f**) VAB. Diamonds indicate input data. Raw (measured) compositional data for basalt are shown as a dashed dark-blue line, and estimated basalt compositional data are shown as a pink line. Primitive mantle data are from Sun and McDonough (1989).

Figure 4 shows examples of PRM estimation for each tectonic setting. These estimations were derived by models using only Th, Nb, Zr, and Ti as input elements. The various compositional patterns of different tectonic settings can be reasonably estimated from these four input elements, within a reproducibility of ± 25%.

### Case study 1: application of the PRMs to seafloor altered basalt

To validate the PRM-reconstructed compositions, we applied the PRMs to seafloor altered basalt using Th, Nb, Zr, and Ti as input elements. The protolith composition of the basalt had been estimated previously from fresh volcanic glass^10^. The reconstructed protolith compositions were then compared with the volcanic glass compositions^10^.

Altered-sample compositions were derived from Ocean Drilling Program (ODP) Site 801^10^ (http://www-odp.tamu.edu/). ODP Site 801 is located in 170 Ma crust to the east of Mariana Island in the Pacific plate. The alteration minerals are commonly saponite and calcite. We applied the PRMs to samples 801-MORB-110-222_ALL and 801C Super, which are characterized by enrichment in Rb, U, K, and Li.

**Figure 5:**
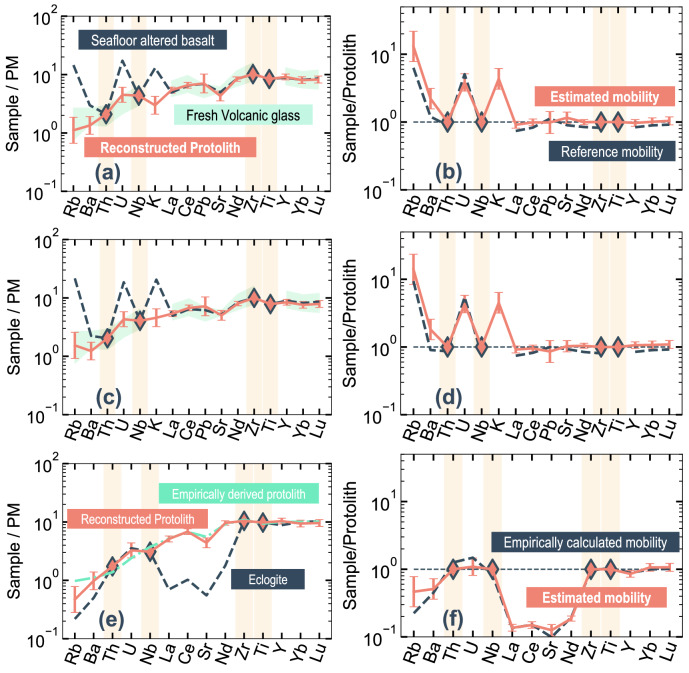
Results obtained using the selected four-element PRMs when applied to seafloor altered basalt and metabasalt, and calculated element mobility. The error bar indicates root mean squared error (RMSE) for each model. Samples in each plot are (**a**, **b**) 801-MORB-110-222_ALL^10^, (**c**, **d**) 801_SUPER, and (**e**, **f**) Z139-6^5^. (**a**, **c**) Primitive-mantle-normalized trace element concentrations estimated for the basalt protolith using the PRMs. Diamonds indicate input data (Th, Nb, Zr, and Ti). Seafloor altered and metamorphic rock compositions are shown as a dashed dark-blue line, and the estimated composition of the protolith basalt is shown as a pink line. The range in protolith compositions derived from fresh volcanic glass is shown as a green region. (**b**, **d**) Calculated element mobility using fresh glass composition (dashed dark-blue line) and estimated protolith (pink line). (**e**) Primitive-mantle-normalized trace element concentrations of estimated protolith basalt. Protolith compositions empirically derived in a previous study^5^ are shown as a green line. (**f**) Calculated element mobility using an empirically derived protolith composition (dark-blue line) and the estimated protolith composition (pink line).

The PRMs were used to reconstruct protolith compositions from altered basalt. The reconstructed protolith compositions have smooth patterns on primitive-mantle-normalized trace element diagrams, and elements with higher compatibility have higher values^56^ (Fig. 5a,c). These PRM-based compositions are within the range of protolith compositions estimated from fresh glass, indicating that protolith compositions can be accurately reconstructed from seafloor basalt.

The element mobility for each sample (Fig. 5b,d) was calculated as follows:

2 $${M}_{i}=\frac{{C}_{i}^{\mathrm{MB}}}{{C}_{i}^{\mathrm{PL}}}$$

where *C*_*i*_^MB^ and *C*_*i*_^PL^ are the concentrations of element *i* in the metabasalt sample and the protolith, respectively. This calculation represents the ratio of element compositions in the altered sample to those in the protolith, thereby removing the protolith contribution and emphasizing the elements affected by mass transfer^10^. Note that the element mobility defined in this study (*M*_*i*_) can be readily converted to the mass change defined by Gresen and Grant^17,18^ which is often used for mass balance analyses (“Supplementary Information Note”). Compared with previous estimates of mobility^10^, results from the PRMs show an accurate estimation of element mobility, ensuring the accurate reconstruction of protolith composition from altered samples or samples affected by mass transfer, within the uncertainty of the estimation (± 0.1 in log_10_ units or ± 25%).

### Case study 2: application to metabasalt and analysis of mass transfer during metamorphism

Using Th, Nb, Zr, and Ti as input elements, we applied the PRMs to an eclogite sample (Z139-6) obtained from central Zambia within the Zambezi belt, part of the Pan-African orogenic system between the Conga and Kalahari cratons^5^. Peak metamorphic conditions have been estimated as 2.6–2.8 GPa and 630–690 °C^58^. The sample is porphyroblastic eclogite composed of omphacite, garnet, kyanite, and quartz that has replaced plagioclase. The sample shows no evidence of prograde blueschist- or amphibolite-facies metamorphism but displays evidence of direct eclogitization from gabbroic assemblages. Reaction textures and chemical analyses have revealed that this sample records prograde eclogitization and mass transfer influenced by fluid derived from the serpentinized lithospheric mantle of a subducting slab^5^. On the basis of comparisons with an empirically determined likely protolith composition, the fluid is inferred to have been strongly undersaturated in light REEs (LREEs) and LILEs^5^. We applied the PRMs to sample Z139-6, which is characterized by depletion in Rb, Ba, La, Ce, Sr, and Nd.

The reconstructed primitive-mantle-normalized protolith concentrations show that elements with higher compatibility have higher values (Fig. 5e). Compared with its protolith, the eclogite is depleted in LREEs (La, Ce, and Nd) and LILEs (Rb, Ba, and Sr). The LREEs and Sr have decreased by about 95%, and Rb and Ba have decreased by 60% and 50%, respectively (Fig. 5f). U and heavy REEs (HREEs) do not show evidence of mass transfer. This pattern of protolith composition and element mobility is consistent with the empirically estimated protolith composition and mass transfer^5^. These results suggest that the PRMs can be used to accurately reconstruct the protolith composition from geochemical data of metamorphic rock.

### Limitations of PRMs in their application to metabasalt

During the application of PRMs to metabasalt, the total mass gain or loss in the sample should be within analytical uncertainty of trace elements (i.e., ± 20 wt%; see “Assumptions of PRMs in their application to metabasalt”). The effects of such uncertainty of the mass gain or loss on the PRM results were evaluated for the seafloor altered basalt considered in Case study I (Fig. S3). As 20 wt% of mass gain or loss results in depletion or enrichment of immobile elements for 20%, we have varied the input element (Th, Nb, Zr, and Ti) concentrations for ± 20%. The resultant PRM-based compositions are still within the range of the protolith composition estimated from the fresh volcanic glass. The reproducibilities of PRMs are within (± 0.1 in log_10_ units or ± 25%) except Rb, for which is within (± 0.2 in log_10_ units or ± 50%). These results suggest that the present PRMs can reasonably reconstruct the protolith composition of metamorphic rocks if the mass gain or loss is within ± 20 wt%.

### Implications of mass transfer analysis based on PRMs

The mass transfer estimated using a PRM is an integral mass transfer from fresh basalt to the analyzed sample (i.e., altered basalt or metamorphic rocks). In the case where an analyzed sample has undergone regional metamorphism, this value includes the mass transfer that occurred during seafloor alteration, prograde metamorphism, and retrograde metamorphism. By utilizing multi-elemental mass transfer data as well as petrological indexes such as reaction extent, these complex mass transfers can be assigned to each geochemical process. A comparison of PRM-based mass transfer with the degree of alteration or retrogression can reveal element transport at a particular stage of alteration or retrogression.

PRMs represent a data-driven method and suffer less bias than protolith estimations reported in previous studies (i.e., based on a geochemist’s experience and intuition). However, caution is needed in applying PRMs to natural samples. First, users need to select input elements that are immobile in the samples of interest. To reasonably assume the immobility of elements, it is necessary to consider previous natural observations, experiments, and the geochemical system for each sample. In cases where the protolith/precursor of samples can be inferred from geological observations, the immobility of elements could also be tested by proportionality of concentrations among potentially immobile elements (i.e., isocon diagrams^17,18^ and/or wedge diagrams^59^). Second, the total mass gain or loss of the sample needs to be reasonably small. To apply PRMs to metabasalt, we infer that the total mass gain or loss in the sample should be within ± 20 wt%, as described in the “Model description” section and in the previous subsection. This is because the current PRMs use element concentrations as input, rather than ratios of concentrations. In the future, PRMs could be improved by using ratios of element concentrations (i.e., Ti/Zr) as inputs to estimate mass gain and loss for the sample compared with fresh basalt.

When these conditions are met, the data-driven approach of the present study is applicable to investigating heterogeneities in protolith composition and provides a less biased and more accurate estimation of metamorphic mass transfer for independent samples compared with previous approaches. Such a data-driven method is suitable for quantitative mass transfer analysis, especially in cases where protoliths are unknown or when there is a need to analyze mass transfer from a compiled dataset with samples from various tectonic settings.

## Conclusion

We developed protolith reconstruction models (PRMs) for metabasalt, using machine-learning with a large basalt compositional dataset. The best PRMs can estimate trace-element compositions of basalt with an error of around ± 0.1 in log_10_ units or ± 25% using only four or five input element concentrations. Using immobile elements as input elements, four-element PRMs were used to estimate protolith compositions of metabasalt. Application to seafloor altered basalt and eclogite verified the accuracy of protolith reconstruction within reasonable uncertainty of the estimation (0.1 in log_10_ units or 25%).

The PRMs used in this study enable the analysis of various types of rock that have undergone mass transfer (e.g., seafloor altered basalt, or rocks affected by contact or regional metamorphism) with the incorporation of appropriate immobile elements. Immobile elements used for PRM inputs can be selected from 511 combinations of 9 elements according to petrological and geochemical observations. Users can select the elements that best suit their application. The machine-learning-based method developed in this study enabled a mass transfer analysis of metabasalt with unknown protolith and can be applied to regional metamorphic belts or alteration zones where the protolith is heterogeneous.

## Methods

The PRMs were constructed using a machine-learning algorithm of the gradient boosting decision tree; specifically, the LightGBM algorithm. To improve empirical model reproducibility, hyperparameters of LightGBM were automatically tuned through Bayesian optimization by using a partial training dataset. Partial training datasets for hyperparameter tuning were prepared by K-fold cross-validation, which enabled us to use all training data in constructing the PRMs. Details of the machine-learning calibrations for PRMs are provided below.

### Gradient boosting decision tree (LightGBM)

Decision tree is a supervised machine-learning method from which prediction models can be constructed from multidimensional data and used to solve classification and regression problems^60^. In the field of geochemistry, this machine-learning method has been applied to extract information, discriminate classes, and predict values; e.g., to discriminate and extract characteristics from a volcanic rock dataset of eight different tectonic settings^26^, classify metamorphic protolith(s) from the major-element composition of a rock^42^, and complement geochemical mapping for improvement of accuracy and interpretation ^61^.

Gradient boosting decision tree (GBDT), one of the decision tree algorithms, has been proposed as explainable models with high accuracy. GBDT is an ensemble method that combines multiple decision trees to build a robust model. In the GBDT method, decision trees are built one after another so that the following decision tree corrects the errors of the previous one^49^. The development of GDBT has enabled various algorithms such as Xgboost^62^ and Catboost^63^ to be proposed, of which LightGBM is an algorithm with fast calculation time and high accuracy^51^. For this reason, LightGBM was used as the machine-learning algorithm and for constructing models to predict element compositions in the present study.

### Tuning hyperparameters

LightGBM is a decision-tree-based nonparametric model. A nonparametric model has a higher degree of freedom than a linear model because of the fewer assumptions needed regarding the training data. However, the flexibility of a decision tree model makes it easier to overfit the training data. To solve this overfitting problem, each model has hyperparameters to restrict the degrees of freedom. The appropriate hyperparameters are dependent on the structure and number of dimensions of the dataset. Accordingly, the hyperparameters need to be optimized for the dataset.

To choose appropriate hyperparameters, we used Bayesian optimization to tune them automatically for the dataset. Bayesian optimization uses the framework of Bayesian probability to select the parameter to be explored based on the history of previously calculated parameters^64^. In this study, Optuna was used as the optimization software^65^, with part of the dataset (termed “validation data”) being used to validate hyperparameter tuning.

The number of hyperparameter searches was set to 50. The tuned hyperparameters and the search space were as follows:

- num_leaves (8–128): the maximum number of leaves in one tree;
- max_depth (2–10): limit the depth for the tree model. This deals with overfitting; and
- min_data_in_leaf (75–500): the minimum number of data in one leaf.

These three parameters are specified in the official LightGBM documentation as the first to be tuned. The other parameters are set with default values.

### Model construction

#### K-fold cross-validation

Data with no missing values in the input and output elements were extracted from the basalt composition dataset and divided into training or test data. One-fifth of the data were used as test data to evaluate the accuracy of the model, and the remaining data were used as training data to construct machine-learning models.

K-fold cross-validation is a way of evaluating the effects of tuning hyperparameters and preventing a reduction in the number of available data (Fig. S4). The training data are randomly split into K distinct subsets. K − 1 subsets are assigned for training the model, and the other subset is used for evaluating the hyperparameters (i.e., validation subset). By changing the subsets used for training and validation, the model is evaluated K times (i.e., K folds)^49^. The average RMSE obtained from all folds is used for hyperparameter tuning by Bayesian optimization. In this study, we constructed a fourfold cross-validation. The reproducibility of the model was evaluated by using the test data (which are independent of the training and validation subsets).

#### Preprocessing of each set of compositional data and Bayesian optimization

To improve the estimation error, input variables are transformed to ratios and products, with a search for the best data representation (i.e., feature engineering). Feature engineering is a common technique for constructing machine-learning models^49^. In this study, we transformed data as ratios and products of concentrations between two arbitrary elements. All of the measured concentration data, ratios, and product were used as preprocessed data for training and construction of the machine-learning models.

Preprocessed training data were used to construct machine-learning models, which were applied to preprocessed validation data to evaluate the reproducibility using the RMSE (Fig. S4). On the basis of the averages of the obtained RMSE values, Bayesian optimization software (Optuna) was used to tune the hyperparameters of the models. We repeated model construction and evaluation 50 times to find the appropriate hyperparameters for each set of compositional data.

## Supplementary Information

Supplementary Information.

## Data Availability

The authors declare that all the necessary geochemical data supporting the findings of this study are available from the references cited in this article (PetDB and the references used in the case studies^5,10^). Any further data are available from the corresponding authors upon request. The python code for PRMs would be available from the corresponding authors upon reasonable request. Authors plan to make the PRMs accessible on web-based applications in the near future.
