# Supplementary material for: Machine-learning techniques for quantifying the protolith composition and mass transfer history of metabasalt
Source: Sci Rep. 2022 Jan 26;12:1385. doi: 10.1038/s41598-022-05109-x (PMC8791977; doi:10.1038/s41598-022-05109-x)
Supplement: Supplementary file 1 — Supplementary Information. [file 41598_2022_5109_MOESM1_ESM.pdf]

Supplementary Information

Title

Machine-learning techniques for quantifying the protolith composition and mass transfer history of metabasalt

Authors

Satoshi Matsuno, Masaoki Uno*, Atsushi Okamoto, and Noriyoshi Tsuchiya

Graduate School of Environmental Studies, Tohoku University, 6-6-20 Aza-Aoba, Aramaki, Aobaku, Sendai 980-8579, Japan

*E-mail: uno@geo.kankyo.tohoku.ac.jp

Supplementary Materials

This **Supplementary Material** contains six Supplementary Figures (Figs. S1–S6), one Supplementary Table (Table S1), and a Supplementary Information Note.

Information Note

Comparison of machine learning algorithms used for constructing PRMs

We compared machine learning algorithms using the fresh basalt dataset used in this study. The input elements were fixed as Th, Nb, Zr and Ti and the output elements were Rb, Ba, U, K, La, Ce, Pb, Sr, Nd, Y, Yb, Lu. For simplicity, we used only the concentrations of the input elements as the input variables, but did not use the ratios and product of these concentrations. The concentrations were normalized to ensure a precise comparison among LightGBM, support vector machine (SVM), multiple linear regression (MLR). As

is the case with the models in the main manuscript, we divided the dataset randomly into training and test data at a ratio of 4:1.

SVM and MLR algorithms were imported from scikit-learn library (version 1.0.1) for python (Buitinck et al., 2013[1]). Regression by SVM was conducted using radial basis function (rbf) kernel.

The results indicate that LightGBM outperformed better than SVM and MLR for all elements (Fig. S5). Fig. S6 indicates that the results of LightGBM have a relatively uniform error distribution, while SVM and MLR have large error variations depending on the estimated concentrations. The average RMSE scores and computational time of all output elements were calculated for each algorithm (Table S1). The average RMSE score is highest for MLR and is lowest for LightGBM. LightGBM outperformed SVM in both calculation speed and reproducibility (Table S1). Therefore, it is optimal to adopt LightGBM among these algorithms.

Relation of the element mobility defined in this study and the mass change by Gresen and Grant

We have evaluated the element mobility based on the method of Kelley et al. (2003) as below:

$$M_i = \frac{C_i^{\mathrm{MB}}}{C_i^{\mathrm{PL}}} \quad (1)$$

where C_i^{MB} and C_i^{PL} are the concentrations of element i in the metabasalt sample and the protolith, respectively. This calculation represents the ratio of element compositions in the altered sample to those in the protolith, thereby removing the protolith contribution and emphasizing the elements affected by mass transfer[2]. In this case, $M_i > 1$ indicates mass gain and $M_i < 1$ indicates mass loss.

Contrary, the mass balance equation given in Grant (1986) is as below[3] (i.e., Gresen's equation):

$$\frac{\Delta m_i}{m^{\mathrm{PL}}} = \left(\frac{m^{\mathrm{MB}}}{m^{\mathrm{PL}}}\right) C_i^{\mathrm{MB}} - C_i^{\mathrm{PL}} \quad (2)$$

where $\underline{m}^{\mathrm{PL}}$ and m^{MB} are the mass of protolith and metabasalt, respectively. Δm_i is the mass change of element i between protolith and metabasalt. In this study, we assume $m^{\mathrm{PL}} \cong m^{\mathrm{MB}}$, thus Eq. 2 can be simplified to $\frac{\Delta m_i}{m^{\mathrm{PL}}} \cong C_i^{\mathrm{MB}} - C_i^{\mathrm{PL}}$. $\Delta m_i > 0$ indicates mass gain and $\Delta m_i < 0$ indicates mass loss. When assuming $m^{\mathrm{PL}} \cong m^{\mathrm{MB}}$, the element mobility ($M_i$) and mass change ($\Delta m_i$) can be converted as below:

$$\frac{\Delta m_i}{m^{\mathrm{PL}}} \cong \Delta C_i = (M_i - 1) C_i^{PL} \quad (3)$$

References

1. Buitinck, L. *et al.* API design for machine learning software: experiences from the scikit-learn project. *arXiv* 1–15 (2013).
2. Kelley, K. A., Plank, T., Ludden, J. & Staudigel, H. Composition of altered oceanic crust at ODP Sites 801 and 1149. *Geochemistry, Geophys. Geosystems* **4**, (2003).
3. Grant, J. A. The isocon diagram-a simple solution to Gresens' equation for metasomatic alteration. *Econ. Geol.* **81**, 1976–1982 (1986).
4. Kadowaki, D., Sakata, R., Hosaka, K. & Hiramatsu, Y. *Data analysis techniques to win Kaggle*. doi:4297108437.

Figures

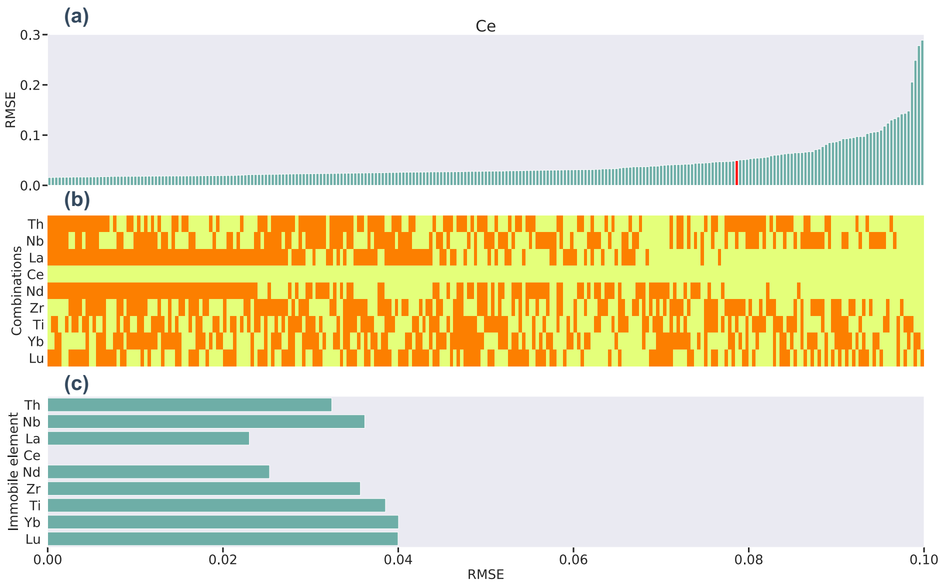

Supplementary Figure S1. (**a**) Average RMSE scores for Ce for each combination of input elements (511 cases), red one indicate the input combination of Th, Nb, Zr, and Ti. (**b**) Combinations of input elements for each model shown in (**a**). Orange elements are used in combinations, and yellow elements are not used. (**c**) Average RMSE scores for Ce of all the models containing a particular element as an input.

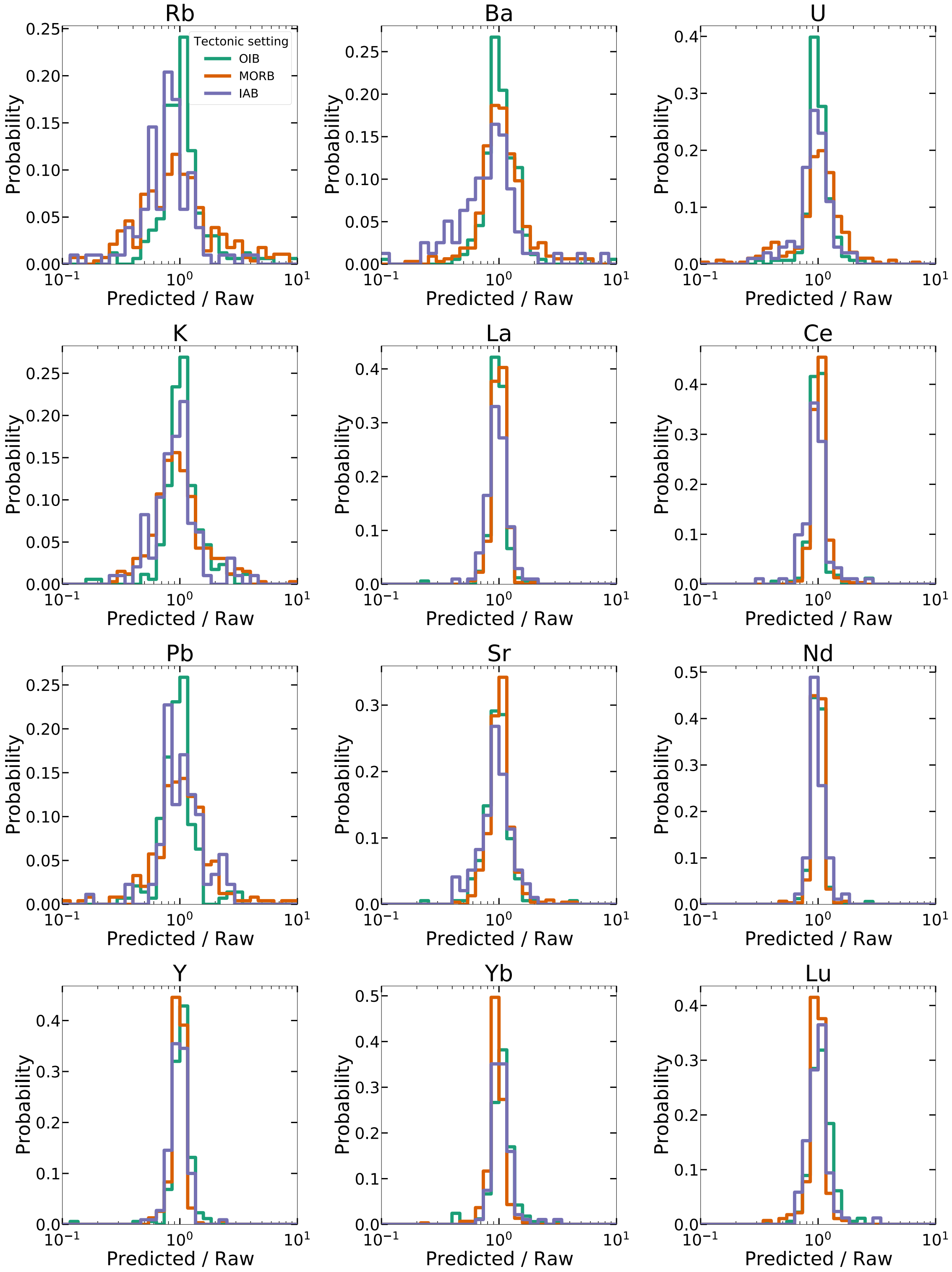

Supplementary Figure S2. Accuracy distribution of test data for each output element using the final PRMs with Th, Nb, Zr, and Ti as input elements. The horizontal axis is the ratio of predicted content to raw (measured) content in log_{10} scale.

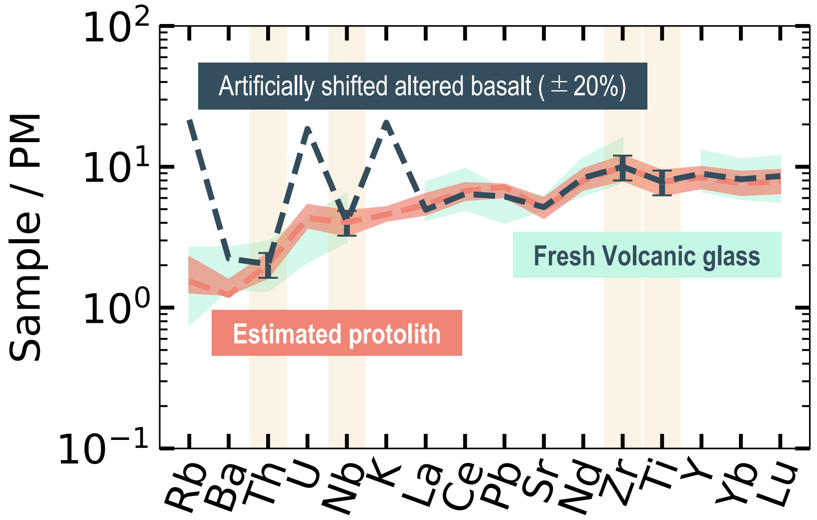

Supplementary Figure S3. Results obtained using the selected four-element PRMs when applied to seafloor altered basalt whose concentrations of input elements (Th, Nb, Zr and Ti) are artificially varied for ±20%. The sample composition (801_SUPER from Kelley et al. 2003)[2] is shown as a dashed dark-blue line, and its error bar indicates the range of the varied input element concentrations (i.e., ±20%). The ranges of the estimated protolith compositions are shown as pink region. The results obtained from the raw data are indicated by dashed pink lines. The range in protolith compositions derived from fresh volcanic glass[2] is shown as green region.

.

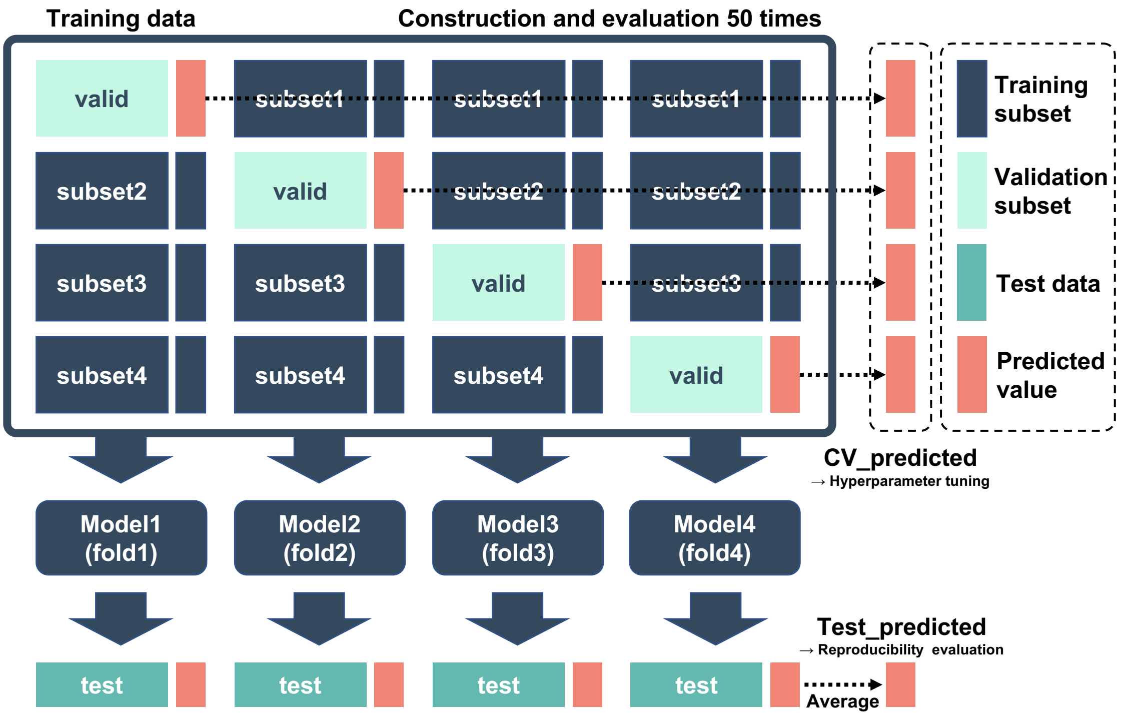

Supplementary Figure S4. Schematic overview of cross-validation. The training data were divided into K subsets. For each fold, $K - 1$ subsets were used to construct machine learning models, and the remaining subsets were used to evaluate the constructed models. The average of the RMSE values obtained from all folds was used for hyperparameter tuning by Bayesian optimization. (Modified after Ref[4]).

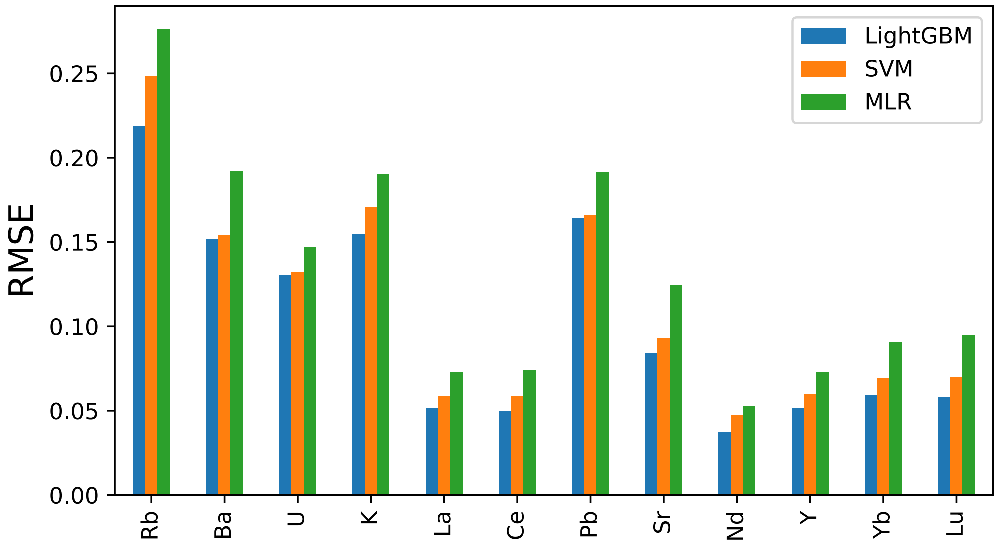

Supplementary Figure S5. RMSEs of LightGBM, SVM, and MLR for each output elements.

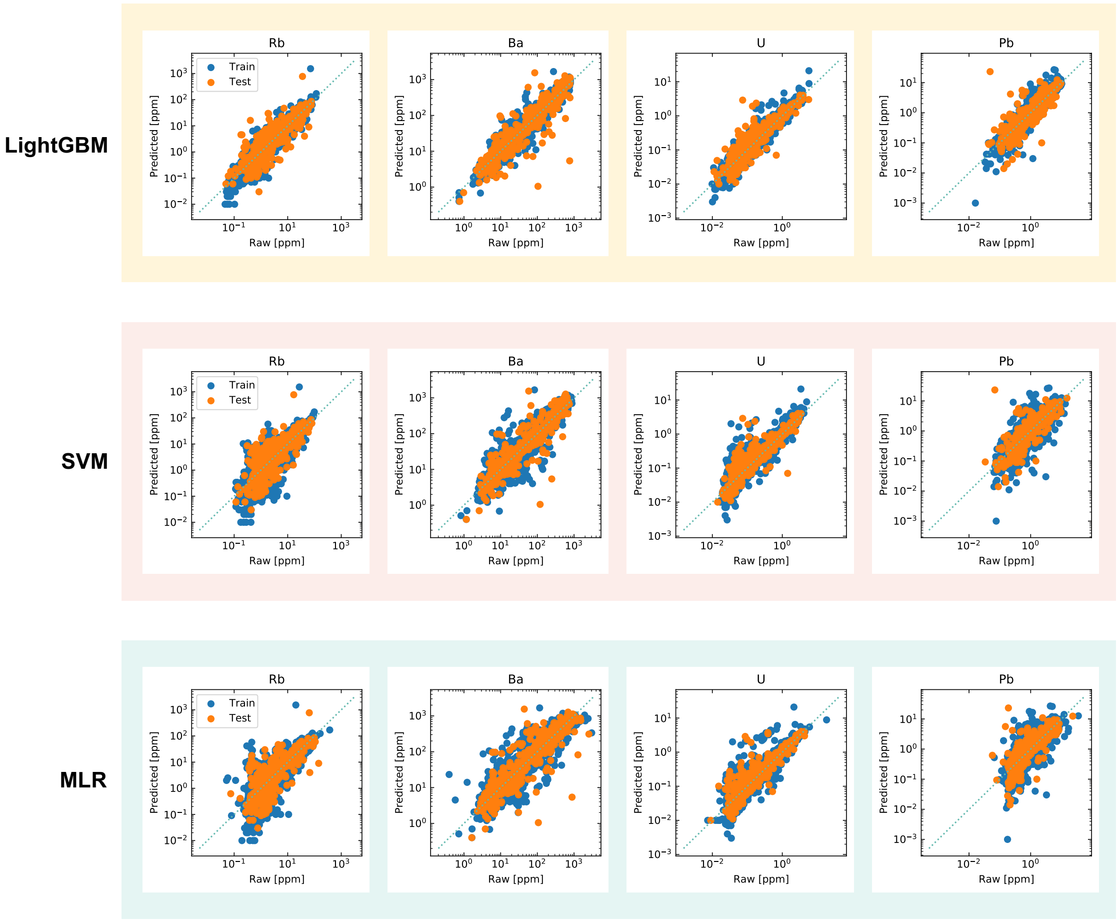

Supplementary Figure S6. Scatter plots of raw (measured) concentrations versus predicted concentrations for Rb, Ba, U, Pb by LightGBM, SVM and MLR.

Tables

Supplementary Table S1. Average performance of all output elements for each algorithm. The performance is evaluated in terms of the computational cost (time), RMSE score for training data (train_score), and RMSE score for test data (test score).

	**Time (sec)**	**train_score**	**test_score**
**LightGBM**	0.107	0.070	0.101
**SVM**	0.185	0.109	0.111
**MLR**	0.001	0.135	0.132
